# DIABEO App Software and Telemedicine Versus Usual Follow-Up in the Treatment of Diabetic Patients: Protocol for the TELESAGE Randomized Controlled Trial

**DOI:** 10.2196/resprot.9154

**Published:** 2018-04-19

**Authors:** Nathalie Jeandidier, Lucy Chaillous, Sylvia Franc, Pierre-Yves Benhamou, Pauline Schaepelynck, Hélène Hanaire, Bogdan Catargi, Anne Farret, Pierre Fontaine, Bruno Guerci, Yves Reznik, Alfred Penfornis, Sophie Borot, Pierre Serusclat, Yacine Kherbachi, Geneviève D'Orsay, Bruno Detournay, Pierre Simon, Guillaume Charpentier

**Affiliations:** ^1^ Department of Endocrinology, Diabetes and Nutrition University Hospital of Strasbourg Strasbourg France; ^2^ Hospital Laennec University Hospital of Nantes Saint-Herblain France; ^3^ Centre d'Étude et de Recherche pour l'Intensification du Traitement du Diabète, Evry Department of Diabetes, Sud-Francilien Hospital University Paris-Sud, Orsay Corbeil-Essonnes France; ^4^ Pôle DigiDune Department of Diabetology University Hospital Grenoble France; ^5^ Department of Nutrition-Endocrinology-Metabolic Disorders, Marseille University Hospital, Sainte Marguerite Hospital Marseille France; ^6^ Department of Diabetology, Metabolic Diseases and Nutrition University Hospital of Toulouse University of Toulouse Toulouse France; ^7^ Department of Endocrinology and Diabetes University Hospital Bordeaux France; ^8^ Department of Endocrinology, Diabetes and Nutrition University Hospital Montpellier France; ^9^ Department of Diabetology University Hospital Lille France; ^10^ Endocrinology-Diabetes Care Unit University of Lorraine Vandoeuvre Lès Nancy France; ^11^ Department of Endocrinology University of Caen Côte de Nacre Regional Hospital Center Caen France; ^12^ Department of Endocrinology, Metabolism, Diabetology and Nutrition University Hospital Jean Minjoz Besançon France; ^13^ Endocrinology, Diabetology and Nutrition Clinique Portes du Sud Venissieux France; ^14^ Sanofi-Diabetes Gentilly France; ^15^ Voluntis Suresnes France; ^16^ CEMKA Contract Research Organization Bourg-la-Reine France; ^17^ National Association of Telemedicine Evry France

**Keywords:** diabetes, diabetes mellitus, telemedicine, eHealth, mHealth, clinical protocols

## Abstract

**Background:**

Self-management of diabetes minimizes the risk of macrovascular and microvascular complications, but understanding and/or adherence to self-management recommendations is often suboptimal. DIABEO is a smartphone app (downloaded via the internet) used to calculate bolus insulin doses. A previous study (TELEDIAB 1) showed that the use of DIABEO was associated with a significant improvement in glycemic control in patients with poorly controlled type 1 diabetes mellitus, particularly when combined with teleconsultations with physicians.

**Objective:**

Here, we present the protocol for a new study (Suivi A Grande Echelle d’une cohorte de diabétiques de type 1 et de type 2 sous schéma insulinique basal bolus par la TELEmédecine; abbreviated TELESAGE), conducted in a larger population of diabetic patients with poorly controlled basal-bolus insulin levels.

**Methods:**

TELESAGE is a multicenter, double-randomized, open-label, three parallel–arms study, conducted in approximately 100 centers in France. The study will compare a control group (arm 1: usual follow-up) with two DIABEO telemedicine systems: (1) physician-assisted telemedicine (arm 2), and (2) nurse-assisted telemonitoring and teleconsultations by a diabetologist’s task delegation (arm 3). Initial randomization will allocate the study arms in 12 French regions. A second randomization will assign patients in the groups allocated to each studied region. The primary objective of TELESAGE will be to investigate the effect of the DIABEO telemedicine system versus usual follow-up, with respect to improvements in the glycated hemoglobin levels of approximately 696 diabetic patients with poorly controlled basal-bolus insulin levels.

**Results:**

The TELESAGE study is sponsored by Sanofi (Gentilly, France). A primary completion date is expected in June 2018, and publication of results is expected within 6 months of work completion.

**Conclusions:**

The TELESAGE study is expected to confirm the previous results of the TELEDIAB 1 study using a larger sample of diabetic patients. It is also expected to evaluate a nurse-assisted telemonitoring system. We will assess the potential of the DIABEO telemedicine service in terms of its utility and explore whether it can become an integral part of diabetes care for patients.

**Trial Registration:**

ClinicalTrials.gov NCT02287532; https://clinicaltrials.gov/ct2/show/NCT02287532 (Archived by WebCite at http://www.webcitation.org/6ykajhJKd)

## Introduction

In its first global report on diabetes, the World Health Organization showed that the number of adults living with diabetes has almost quadrupled since 1980 to 422 million adults [1]. Diabetes complications can lead to blindness, heart attack, renal insufficiency, stroke and lower limb amputation. In 2012, diabetes caused 1.5 million deaths [1].

Self-management of diabetes is crucial to minimize the risk of macrovascular and microvascular complications [2,3]. This involves a daily planning of diet and physical activity, proper use of prescribed medication, and self-monitoring of capillary blood glucose levels. All this is done in order to adjust diet, physical activity and insulin treatment. However, adherence to self-management recommendations is often suboptimal, which is of importance for people whose diabetes is poorly controlled [4].

The Scottish registry linkage study [5] shows that only 13%–15% of patients with type 1 diabetes mellitus (T1DM) meet the glycated hemoglobin (HbA_1c_) target level of less than 7.0% [5], whereas more than 20% have very poor glycemic control (HbA_1c_>8.8%). The hazard ratios for death from cardiovascular causes increase from 2.9 in well controlled patients to 10.5 in the poorly controlled ones [6]. The reasons for the insufficient glycemic control among T1DM patients are numerous. T1DM is a complex, relatively infrequent disease that is managed by a diabetologist. The complex rules for calculating insulin doses can lead T1DM patients to inject inappropriate doses, especially during meals, leading to episodes of hypo- or hyperglycemia.

Patients with type 2 diabetes mellitus (T2DM) under intensive basal-bolus insulin regimens face similar problems [7]. An automatic system calculating bolus insulin doses on a daily basis is necessary to help both T1DM and T2DM patients undergoing an intensive insulin regimen. On the other hand, the extreme burden of daily routine (eg, glycemic control, carbohydrate counting, and determining an insulin dose that takes into account additional parameters such as irregular activities or unexpected physical activity) can be reduced through telemedical health care team support when needed. Telemedicine may also help intensively treated T1DM or T2DM patients, often those who are young and/or actively working, who find it difficult to comply with scheduled doctor visits to avoid progressive diabetes control degradation). It is essential for these patients to rapidly contact their caregiver, if necessary by telephone and email. Finally, alerts may help caregivers reach patients when needed.

The DIABEO system was created to overcome some of the above hurdles [8,9]. DIABEO is an app for insulin dosage calculation, available for download on smartphones. It calculates bolus insulin doses according to medical prescription and uses validated algorithms to take into account the carbohydrate intake, predrug glucose and anticipated physical activity reported by the patient. It provides glycemic targets and automatic algorithms for the adjustment of carbohydrate and basal insulin or basal pump rates when plasma postprandial or fasting glucose levels are off target. An internet connection ensures data transmission by means of automatic messages to medical staff (through a secure connection and website) to facilitate remote monitoring and teleconsultations.

In 2009, a pilot study demonstrated the feasibility, safety and accuracy of DIABEO [8]. Moreover, a six-month, open-label, randomized clinical trial conducted in 180 poorly controlled T1DM patients (TELEDIAB 1 study) showed that the DIABEO software combined with short teleconsultations (ie, five minutes every two weeks) demonstrated a 0.91% improvement in HbA_1c_ over controls and a 0.67% reduction when the DIABEO software is used alone [9]. This benefit does not require more medical time and is obtained at a lower overall cost for the patient than usual care [9].

Following the TELEDIAB 1 study, the Haute Autorité de Santé, France (HAS) approved DIABEO as a medical device for use in T1DM patients (July 2016) [10]. DIABEO was approved for two years, and the HAS specified that the renewal will be conditioned on the results of the current study (*Suivi A Grande Echelle d’une cohorte de diabétiques de type 1 et de type 2 sous schéma insulinique basal bolus par la TELEmédecine*; abbreviated TELESAGE).

The purpose of TELESAGE is to investigate the metabolic efficacy of the DIABEO telemedicine service in a large population of patients with poorly controlled diabetes who are on a basal-bolus insulin regimen. Additionally, we will assess its economic impact in terms of cost reduction to the health insurance system. Here, we present the protocol of the TELESAGE study.

## Methods

### Objective

TELESAGE was designed to investigate the efficacy of the DIABEO telemedicine service in improving glycaemic control in a large population of diabetic patients sub-optimally controlled with insulin.

### Study Design

**Figure 1:**
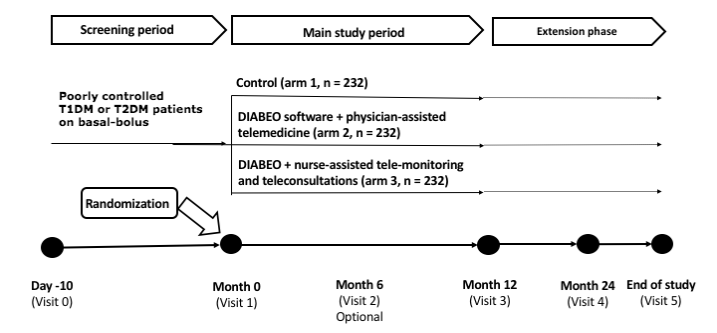
Study design. T1DM: type 1 diabetes mellitus; T2DM: type 2 diabetes mellitus.

TELESAGE is a randomized, open-label, three parallel-arms study that is to be conducted in approximately 100 public and private centers that employ diabetologists in France (Figure 1). The study protocol was designed by Centre d'Étude et de Recherche pour l'Intensification du Traitement du Diabète (CERITD; Evry, France). CERITD is a nonprofit clinical translational research center located in Corbeil Hospital (Corbeil-Essonnes, France). Selected centers have been already participating in the TELEDIAB 1 study [9]. Voluntis (Suresnes, France) provided the DIABEO software, Orange Telephone Company (Paris, France) provided the smartphone and telephone lines, and Sanofi (France) funded the study.

The study was designed to include a population of approximately 696 T1DM and T2DM patients poorly controlled with a basal-bolus insulin regimen (HbA_1c_≥8%) in real-life conditions. The patient recruitment period was estimated to last approximately 36 months.

The trial compares a control group (arm 1: usual follow-up) with the previously investigated DIABEO telemedicine service (arm 2: software + physician-assisted telemedicine as in the TELEDIAB 1 study [9]) or a new DIABEO telemedicine service (arm 3: software + telemonitoring and teleconsultations delegated by the diabetologists to a nursing staff) (Figure 1). Participants are asked to carry out at least two self-monitoring plasma glucose (SMPG) every day during the study. Patients randomized to arms 2 and 3 receive a smartphone with the DIABEO software. The investigator-physician fixes glycemic targets and associated treatment, alarm values, and values for self-adaptations. Patient enters daily three types of variables in the app: (i) SMPG levels before and after meals (6 measurements) + 1 optional in the night; (ii) carbohydrate counts; and (iii) planned physical activity. Patient entry data is automatically uploaded by the smartphone to a secured website (available to investigators at any time). If fasting or postprandial SMPG do not meet target levels, the system can suggest adjustments for carbohydrate ratio, long-acting insulin analog dose, or pump basal rates.

Following a screening period of 10 days, the main study period will last 12 months, with an optional extension period of at least 12 additional months (Figure 1). If desired, patients from the control group can begin to use the software after 12 months.

#### Physician-Assisted Telemedicine (Arm 2)

Teleconsultations will be conducted with both patients and doctors in front of their computers or smartphone displaying data from the week before. These sessions focused on insulin dose adjustments and motivational support.

#### Physician/Nurse Delegation Protocol (Arm 3)

**Figure 2:**
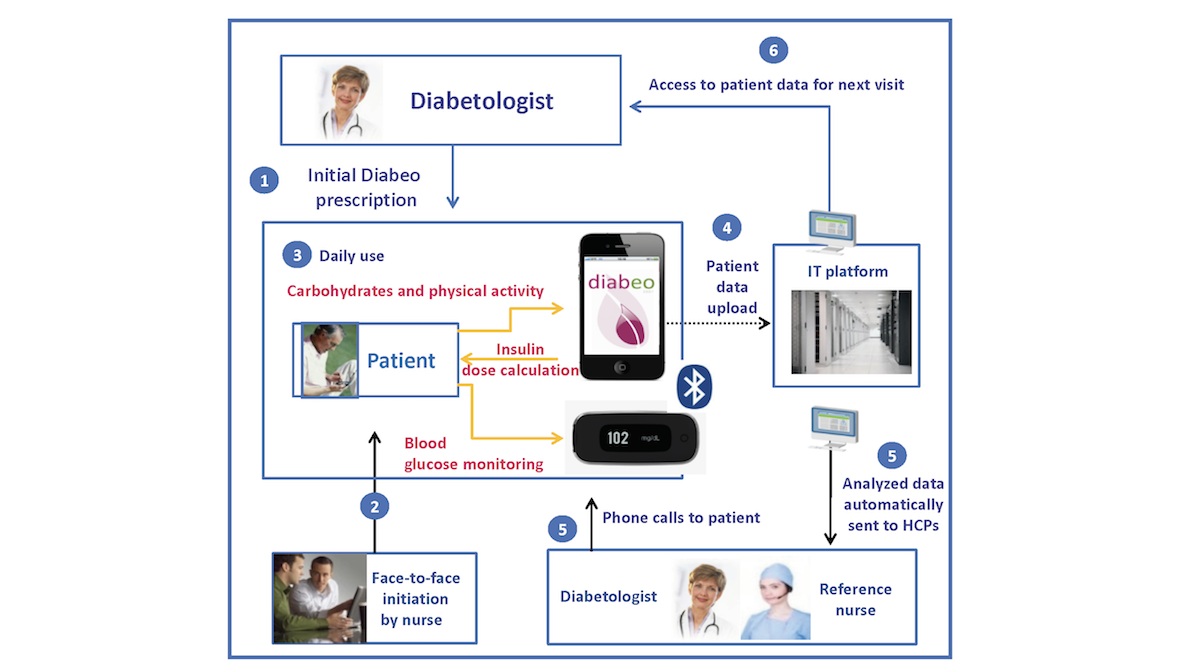
Road map of arm 3. Patient enters daily three types of variables in the DIABEO application (step 3): (i) Self-measured plasma glucose levels before and after meals (6 measurements) + 1 optional in the night; (ii) carbohydrate counts; and (iii) planned physical activity (see text for other technical details). HCP: health care practitioner.

Arm 3 of the study involves a nursing team supervised by a physician, based in Paris and its surrounding region. Figure 2 shows the road map of arm 3, which starts with the investigator-physician, who fixes glycemic targets and associated treatment, alarm values triggering a nurse action, and values for self-adaptations (step 1). A reference nurse performs patient initiations (step 2). The patient can now use the DIABEO app on their smartphone (step 3). The device performs a titration of the insulin dose (and eventually a proposal for dose adaptation) as a function of several factors, including blood glucose levels, physical activity and ingested carbohydrates. The data entered by the patient is sent to a secure platform every 2 hours (step 4). This platform is continuously visible to the referring nurse and the diabetologist. Automatic messages containing analytical data are generated every night (step 5). The referring nurse (who can call the patient and/or the diabetologist, if necessary) analyzes these messages during the morning of each working day. Finally, the diabetologist receives patient data and nursing reports (step 6).

#### DIABEO Software

The DIABEO software was described in the *Introduction* section (for details, see references [8,9]). Telemedicine is similar to that previously described [9], with the exception that patient teleconsultations in arm 3 are conducted by nurses instead of doctors.

#### Clinical Study Flow Diagram

The schedule of visits and measurements is given in Table 1. HbA_1c_ measures assessing glycemic control are performed at visits 1, 2 (optional), 3 and 4.

### Ethical Conduct of the Study and Informed Consent

The study is conducted in accordance with the Declaration of Helsinki and Good Clinical Practice guidelines and in accordance with French privacy law (*Informatique et Libertés*) when processing personal data in the health care field (Act of 6 January 1978, amended by Law No 2004-801 of August 6, 2004).

This clinical trial began after the sponsor had obtained approval from the ethical committee (*Comité de Protection des Personnes* [CPP]; Committee for People Protection) of La Pitié-Salpetrière Hospital (Ile de France VI) and the authorization of the French *Agence Nationale de Sécurité du Médicament* (ANSM; National Agency for Drug Safety). The study was registered under ANSM# 2012-A00072-41. The sponsor communicates all serious and unexpected adverse events to the CPP and the ANSM.

Before inclusion, patients consented to participate in the trial. To that purpose, patients were informed about the nature, objective and possible consequences of the trial, and gave signed consent to participate and release medical-related data.

### Patients

As calculated in the Statistical Analysis section, 696 subjects have been included in the study (the recommended number of subjects per center was 6-8 patients, the inclusion period lasted from April 24, 2013 to May 19, 2016). The trial is currently active, but not completed.

**Table 1:** Clinical study flow diagram; schedule of enrollment, interventions and assessments. EQ-5D: EuroQol five dimension scale; HbA_1c_: glycated hemoglobin.

Conponents	Visit #1	Visit #2	Visit #3	Visit #4^a,b,c^	Visit #5^d^ (Optional extension)
Evaluations	Day 0	Month 6	Month 12	Month 24	End of study
Informed consent	✓		✓^e^	✓^d^	
Inclusion / exclusion criteria	✓				
Medical history	✓				
Demographics	✓				
Concomitant treatments	✓	✓	✓	✓	✓
Clinical examination	✓	✓	✓	✓^a,f^	
Randomization	✓				
Weight	✓	✓	✓	✓	✓
Blood pressure	✓	✓	✓	✓^a,f^	
Last HbA_1c_ values	✓	✓	✓	✓	✓
Questionnaires (EQ-5D)	✓		✓	✓^a,f^	
DIABEO initiation	✓^a,b,f^		✓^c^		
Nursing appointment^a^	✓		✓^e^		
Fixing appointment for visit #3		✓			
Satisfaction questionnaire “DIABEO”			✓^a,f^	✓^a,f^	
Severe hypoglycemia		✓	✓	✓^a,f^	
Symptomatic hypoglycemia (≤15 days before)			✓		
Adverse events	To be reported all throughout the study				
Serious adverse events	To be declared to sponsor within 24 hours (next business day)				
Malfunction of the DIABEO software^f^	To be reported all throughout the study				
Care consumption	To be reported every month by all concerned patients				
Remittance of TELESAGE books	✓		✓^a,f^		

^a^Applicable to group 3 (software + nurses’ telemonitoring and teleconsultations).

^b^Initiation takes place within approximately 10 days after the inclusion visit.

^c^Applicable to patients of group 1 (control) continuing the study after 12 months and using the software.

^d^Applicable to patients wishing to use DIABEO during the extension phase and who have not yet signed a consent for that purpose.

^e^Applicable to patients of group 1 (control) continuing the study after 12 months and using the software + nurses’ telemonitoring and teleconsultations.

^f^Applicable to group 2 (software + physicians’ telemedicine).

### Inclusion Criteria

Patients enrolled in the TELESAGE study should meet the following inclusion criteria: T1DM and T2DM patient performing self-monitoring of blood glucose (≥2 measured values per day), treated with insulin analogs according to a basal-bolus regimen for at least 1 year and using the same method of administration (pen or pump) for at least 3 months, possessing an Apple or Android smartphone compatible with DIABEO before starting the study, having two HbA_1c_ values ≥8%, one dating to more than 3 months ago and the other less than 1 month ago. Additionally, participants must have the capability to understand and follow the instructions of the study, and be able to provide written consent to participate and specify if they benefit from a social security scheme.

### Exclusion Criteria

Key exclusion criteria included: age <18 years; subject having already used the DIABEO system in the 6 months preceding inclusion, or participating in a clinical trial within 6 months (except Meos, the TELEDIAB 3 study after a 6-month participation period), or treated with human insulin, or pregnant (or wishing to be pregnant during the study period), or subject requiring boluses >0.4 IU/gram (for subjects under functional insulin therapy) or >99 IU per day (for subjects treated on a fixed diet plan), or subject living with staggered hours (eg, night work, meals shifted).

### Randomization

Patient randomization is automatically done by using the electronic case report form software. A first randomization step allocates the study arms at the regional level: (i) six regions including patients in arms 1 and 2 (Aquitaine, Île-de-France, Lorraine, Nord-Pas-de-Calais, Rhône-Alpes, and Languedoc-Roussillon) and (ii) six other regions including patients in arms 1 and 3 (Alsace, Franche-Comté, Lower Normandy, Midi-Pyrénées, Pays de la Loire, and Provence-Alpes-Côte d'Azur). Then, within each region the patients are further randomized between the two groups (done at patients’ inclusion, between arms 1 and 2 or between arms 1 and 3). The distribution by center was 1:2 (ie, 1 patient of arm 1 for 2 patients of arms 2 or 3).

### Outcome Measures

The primary outcome measure of this study is to investigate the effect of a 12-month follow-up with the DIABEO system (software + physicians’ telemedicine, or software + nursing telemonitoring, and teleconsultations by diabetologist’s task delegation) versus usual follow-up in terms of improvement of glycemic control (HbA_1c_ levels) in T1DM or T2DM patients poorly controlled by a basal-bolus insulin regimen. HbA_1c_ high performance liquid chromatography assays are performed at qualified medical biology laboratories and then reported by participants to investigators. Secondary outcome measures are to compare groups for: HbA_1c_ levels, percent of responder patients (HbA_1c_ <7.5% or HbA_1c_ reduction ≥1%) and severe hypoglycemia at 6, 12 and 24 months, as well as for quality of life and satisfaction (of patients and physicians) at 12 and 24 months. Severe hypoglycemia was defined as requiring third-party assistance. Quality of life was assessed by a specific questionnaire, derived from the EuroQol five dimension scale (EQ-5D) questionnaire [11]. For participants of arms 2 and 3, satisfaction with the DIABEO telemedicine system is evaluated at each center, with a patient’s self-assessed specific questionnaire.

Other secondary, medico-economic outcome measures, have been designed to compare groups at 12 and 24 months for resource consumption and health insurance costs (including overall costs of diabetes and complications, and costs per point of HbA_1c_ reduction and for severe hypoglycemia avoided). If the study demonstrates an overall statistically significant effect, subgroups analyses will be conducted to identify the patients’ profiles with optimal costs consequences and cost-effectiveness ratios.

### Statistical Analysis

#### Patient Population Size

The initial sample size to detect a ≥0.5% difference in HbA_1c_ from baseline to month 12 was estimated using a standard deviation of 1.2%, a rate of not evaluable patients of 15% and an intracluster correlation coefficient of .005 (a measure degree of homogeneity within the same region). This calculation predicted an initial sample size of 696 patients to achieve ≥90% power in detecting a difference in outcome.

#### Data Analysis

Efficacy outcomes are analyzed on an intention-to-treat basis. A confirmatory analysis adjusted by center and region will be carried out and a robustness analysis will be done on the population per protocol. Categorical data are expressed as frequencies and percentages, while quantitative data are expressed as means and standard deviations. The analysis of covariance (ANCOVA) is used to compare groups for the results on the primary end point. The ANCOVA, the chi-square test, and Fisher exact test are used for other comparisons.

## Results

The TELESAGE study is sponsored by Sanofi (Gentilly, France). A primary completion date is expected in June 2018, and publication of results is expected within 6 months of work completion.

## Discussion

### Study Rationale

The DIABEO telemedicine system has previously showed superiority to usual follow-up in improving HbA_1c_ in patients with poorly controlled diabetes [9]. The current TELESAGE study is expected to confirm this result in a larger sample of diabetic patients and real-life conditions. Moreover, the TELESAGE study will validate the present physician/nurse delegation protocol.

The TELEDIAB 1 study showed that the DIABEO telemedicine system improves HbA_1c_ without requiring more medical time and providing far more services compared to usual care [9]. In this respect, the TELESAGE study will test the efficacy of a closer follow-up by the nursing staff as compared with the previous physician-assisted telemedicine system.

Usually, a T1DM or T2DM patient undergoing a basal-bolus insulin regimen sees their diabetologist every 3 to 6 months. At the hospital, the patient can also be monitored by a nurse for therapeutic education (whether or not the patient is hospitalized), which can help in difficult moments (eg, hospitalization following a serious adverse event or during a major treatment switch such as from injectable treatment to insulin pump treatment).

The DIABEO telemedicine service has several strengths. Patient data is daily analyzed by the DIABEO system to fight against glycemic instability and complications. In very serious cases, analysis of these data allows triggering nursing actions and/or physician actions. Nursing delegation also allows more availability to receive patients’ calls and respond to daily issues. Continuity and continuity of care is thus organized from Monday to Saturday from 8am to 8pm.

If positive results are obtained, TELESAGE will clearly demonstrate that the DIABEO telemedicine service could be an integral part of the ambulatory care of an insulin-treated patient.

### Limitations

Given the impossibility of double-blind assessment of open-label intervention versus usual follow-up, the effects of report bias cannot be eliminated.

## Abbreviations

ANCOVA: analysis of covariance
ANSM: Agence Nationale de Sécurité du Médicament (National Agency for Drug Safety)
BMS: Bristol-Myers Squibb
CERITD: Centre d'Étude et de Recherche pour l'Intensification du Traitement du Diabète
CPP: Comité de Protection des Personnes (Committee for People Protection)
EQ-5D: EuroQol five dimension scale
GSK: GlaxoSmithKline
HAS: Haute Autorité de Santé (France)
HbA_1c_: hemoglobin A_1c_
MSD: Merck Sharp and Dohme
SMPG: self-monitoring plasma glucose
T1DM: type 1 diabetes mellitus
T2DM: type 2 diabetes mellitus
TELESAGE: Suivi à Grande Échelle d’une cohorte de diabétiques de type 1 et de type 2 sous schéma insulinique basal bolus par la TELEmédecine
